# 

**Published:** 1892-06-25

**Authors:** 


**Notices of Births, Deaths, and Marriages,** are inserted in this column at a charge of 2s. 6d. for an announcement not exceeding four lines.

### NOTICE TO CORRESPONDENTS.

All MS., Letters, Books for Review, and other matters intended for the Editor should be addressed The Editor, The Lodge, Porchester Square, London, W.

The Editor cannot undertake to return rejected M.S., even when accompanied by stamped directed envelope.

### Notice to Advertisers and Subscribers.

All Advertisements, Orders for Copies of The Hospital, and Business Communications should be addressed to The Publisher, *not to the Editor*, The Hospital, 140, Strand, W.C.

The Cost of Subscription, post free, is as follows:—Per week, 2½d.; per quarter, 2s. 6d.; per half-year, 5s.; per annum, 8s. 6d. Foreign:—Per annum, 10s. 8d.; payable, in all cases, in advance.

### "Burdett's Hospital Annual," 1891—1892.

Third year of publication. 600 pp. Boards, gilt lettering. A most complete guide to British and Colonial Hospitals, Dispensaries, Nursing and Convalescent Institutions, and Asylums. Price 3s. 6d. Published by The Scientific Press (Limited), 140, Strand, W.C.

**NOTICE.—The Index for Vol. XI. (ending March, 1892) is on sale at the Office of this Journal, price 2½d., post free.**

### SCRAPS AND GLEANINGS.

THE total amount of the Ripon Hospital Sunday Fund amounted to £22 12s 7d.

DURING the year 1891 there were 1,230 cases of scarlet fever admitted into the City Fever Hospital, Birmingham.

PRINCESS CHRISTIAN recently visited the Windsor Royal Infirmary for the purpose of opening a new children's ward.

THE Luton Hospital Saturday Fund resulted in £189 17s. 6d¼., which was given to the general fund of the hospital.

LAST Sunday the Duke of Connaught inspected the Corps of Commissionaires, at the Royal Hospital, Chelsea.

THE Gordon Boys' Home has received about £230 as the result of the concert held in St. James's Hall in aid of that institution.

SURGEON CAPTAIN H. E. DEANE has been appointed to the charge of the Hospital for Soldiers' Wives and Children at Aldershot.

THE total amount realised by the collections in Luton on Hospital Saturday amounted to £60, being more than treble the sum collected on former occasions.

MRS. RUTTER has died at Cambridge from poison, having used in cooking a root of what she believed to be horse-radish, but which was afterwards found to be nightshade.

THE Hon. W. F. D. Smith, M.P., and Mr. W. L. A. Burdett-Coutts, M.P., have been elected members of the Committee of Management of the Royal Westminster Ophthalmic Hospital.

THE three days' Bazaar and Fancy Dress Fair in aid of the building fund of a cottage hospital for Willesden, was opened last week by the Countess of Aberdeen, at the Hampstead Conservatoire.

PRINCESS CHRISTIAN OF SCHLESWIG HOLSTEIN has consented to open the Lady Mayoress's rose show and floral fête, to be given in aid of the funds of the Royal Hospital for Children and Women, at the Mansion House, on Friday, the 24th inst.

PRINCESS CHRISTIAN last week laid the foundation-stone of the building which is to replace the old Home for Working Boys in Spital Square. About £4,150 has already been received towards the £5,000 needed for the new building.

THE new buildings in connection with the Coldash Children's Cottage Hospital are two storeys in height, and fitted for twenty beds. The site was given by Mr. Allen Graham, of London. Mr. James H. Money, of Newbury, is the architect. There is a deficiency of £341 on behalf of the building fund.

SIR SYDNEY HEDLEY WATERLOW, who has filled the office of Treasurer of St. Bartholomew's Hospital since 1874 has intimated to the Governors that he does not propose to offer himself for re-election this year. After fifty-five years of work in the City he is recommended to pass the winter in the Riviera.

WILL all kind friends who, during the summer months, send boxes of cut flowers to the London hospitals, remember that when properly packed these flowers give the greatest possible pleasure to many a poor suffering patient; whereas, if they are loosely packed in old boxes, they usually arrive withered and useless.

MRS. CALVERLY BEWICKE will open Queen Anne's Mansions New Theatre on the 25th inst., with a Shakesperian recital, in aid of the Samaritan Fund of the Westminster Hospital. Archdeacon Farrar will preside. This Fund provides patients on leaving the Hospital with that clothing which some of them so sorely need.

LAST Sunday being Hospital Sunday, the Lord Mayor, the Sheriffs, and many members of the Corporation attended the morning service in Westminster Abbey and the afternoon service at St. Paul's. The offertory at Westminster Abbey amounted to £214 14s. 2d., and at St. Paul's to about £230. The Temple Church contributed £197 1s. 2d.

THE Convalescent Home of the Chelsea Hospital for Women, at St. Leonards-on-Sea, which was opened by Lady Cadogan a year ago, is now in full working order, and doing excellent service. The building and furnishing expenses have been entirely defrayed by generous donors, but subscriptions and donations are much needed to carry on the work.

THERE can be but little doubt, the *Lancet* says, that cholera is spreading in Persia in a direction and on a scale that is likely to constitute a grave danger to Europe. It is following the old route by which cholera has travelled from East to West in former years. Hence other countries in Europe besides Russia should be prepared for any emergency that may arise.

AT the annual meeting of the Dundee Royal Infirmary the Secretary stated that the total number of cases treated during the year ended on May 15th was 2,140. The average number of patients in the house each day throughout the year was 159, the highest number on any one day 172. The work in connection with the electric lighting and steam laundry machinery have now been completed, the result proving most satisfactory.

AT a special meeting of the Hastings Hospital, the following alteration of rule No. 53 was proposed and carried: "No person shall be elected to the office of Physician or Assistant Physician unless he be a Fellow or Member by examination of the College of Physicians, London, Edinburgh, or Dublin, and be a Graduate in Medicine of one of the universities of the United Kingdom, and be also duly registered under the Medical Acts."

THE foundation stone of St. Leonard's Hospital, Bedford, was laid by Lord Ampthill. It is to be in the old English style of architecture, half timbered with rough cast between studs and consists of a large room, 63 ft. by 22 ft., with lofty open roof, intended for entertainments; waiting-room, 23 ft. by 13 ft.; long-room or kitchen, 16 ft. by 12 ft. 6 in. There are also three bed-rooms and the usual out offices. The total cost of the building is to be about £1,000.

### THE STUDENT OF MEDICINE.

[All communications for this Supplement should be addressed to THE EDITOR of THE HOSPITAL, at 140, Strand, with the words, "Students' Column," written in the left hand top corner of the envelope.]

### APPOINTMENTS.

Collum, Archie J., L.R.C.P., M.R.C.S., appointed Surgical Registrar to Charing Cross Hospital. Colman, Walter S., M.B., M.R.C.P., appointed Registrar and Pathologist to the Hospital for Sick Children, Great Ormond Street. Devereux, William C., B.A.Cantab., L.R.C.P.Lond., M.R.C.S.Eng., appointed Honorary Surgeon to the Tewkesbury Hospital. Foster, M. B., M.R.C.S., L.R.C.P., appointed House Surgeon to Charing Cross Hospital. Maclean, Ewen J., M.D., C.M.Edin., appointed Resident Medical Officer to the Chelsea Hospital for Women. O'Sullivan, Patrick T., M.B., B.Ch., B.A.O. (Royal University of Ireland), L.A.H.Dub., F.C.S., appointed House Surgeon and Apothecary to the South Charitable Infirmary and County Hospital, Cork. Palmer, W. M., L.S.A., L.R.C.P., M.R.C.S., appointed Resident Obstetric Officer to Charing Cross Hospital. Senior, H. D., M.R.C.S., L.R.C.P., appointed House Physician to Charing Cross Hospital. Smith, Percy, M.R.C.S., L.R.C.P.Lond., L.D.S., appointed Dental Surgeon to the Victoria Hospital for Children, Queen's Road, S.W. Swain, James, M.D., M.S.Lond., F.R.C.S.Eng., appointed Honorary Assistant Surgeon to the Bristol Royal Infirmary. Tawes, G. W. H., M.B., C.M.Aber., late House Surgeon, Royal Infirmary, Aberdeen, appointed Junior Assistant Medical Officer to the Counties Asylum, Carlisle.

### VACANCIES.

The following vacancies are announced this week, the amount of salary in each case being given after the name of the institution:

### Resident Medical Officers.

Belgrave Hospital for Children, 79, Gloucester Street, S.W., House Surgeon—Board, &c.
Bridgnorth and South Shropshire Infirmary, House Surgeon—Salary, £80 per annum; rising £10 annually to £100, with board, &c.
East London Hospital for Children, Glamis Road, Shadwell, E., House Surgeon—Board, &c.
East London Hospital for Children, Glamis Road, Shadwell, E., House Physician—Board, &c.
General Hospital, Birmingham, Assistant House Surgeon—Board, &c.
General Hospital, Birmingham, Resident Surgical Officer; doubly qualified—Salary, £130 per annum, with board, &c.
Hospital for Consumption and Diseases of the Chest, Brompton, Resident House Physicians.
Royal South Hants Infirmary, Southampton, Assistant House Surgeon—Board, &c.
Victoria Hospital for Children, Hull, Resident Medical Officer—Salary, £50 per annum, with board, &c.
Warneford Hospital, Leamington, Spa, House Surgeon—Salary, £100 per annum, with board, &c.
Wolverhampton and Staffordshire General Hospital, Wolverhampton, House Physician—Salary, £100 per annum, with board, &c.
York Dispensary, Resident Medical Officer, unmarried—Salary, £150 per annum, with furnished apartments, &c.

### Non-Resident Medical Officers.

Belgrave Hospital for Children, 79, Gloucester Street, S.W., Dental Surgeon.
Dental Hospital of London, Leicester Square, W.C., Assistant Dental Surgeon; must be L.D.S.
Dental Hospital of London and London School of Dental Surgery, Leicester Square, Demonstrator—Honorarium £50 per annum.
Royal Westminster Ophthalmic Hospital, King William Street, West Strand, Clinical Assistants—Appointment for six months.
St. Marylebone General Dispensary, 77, Welbeck Street, Cavendish Square, Honorary Physician.
Westminster Hospital, Broad Sanctuary, S.W., Assistant Ophthalmic Surgeon; must be F.R.C.S.Eng.

### PASS LISTS.

### Royal College of Surgeons, Edinburgh.

The following gentlemen having passed the requisite examinations, have been admitted Fellows of the College since last publication: A. E. Davis, L.R.C.S.Edin.; J. Bark, M.R.C.S.Eng.; C. E. Bean, M.R.C.S.

### THE STUDENT OF MEDICINE—(continued).

Eng.; W. T. Crawford, L.R.C.S.Edin.; and J. A. Greig, L.R.C.S. Edin. S. Snell, M.R.C.S.Eng., was admitted a Fellow of the College without examination.

The following gentlemen having passed the requisite examinations, were admitted Licentiates of the College: E. Kingscote, D. C. M. Lunt, W. E. Lamond, W. I. Fern, J. Crooks, F. N. Burwell, J. C. G. Macnab, F. J. Spilsbury, and J. A. Menzies.

The following gentlemen having passed the requisite examinations, received the Diploma in Public Health of the College: F. W. Thomson, M.B., C.M., B. G. Brock, L.R.C.P. and S.E., A. B. Cottell, F.R.C.S.E., T. H. Littlejohn, M.B., C.M., S. J. R. Greville, M.R.C.S.Eng., and Louis Demetriadi, F.R.C.S.E.

The following gentlemen having passed the requisite examinations, were admitted Licentiates in Dental Surgery of the College: W. Guy, F. J. Turnbull, W. Williams, and T. E. Johnston.

The Pattison Fund Prizes in Practical Anatomy have been awarded by the College for the year 1891-2 as follows: First prize (£25), Miss Elsie Maud Inglis; second prize (£10), Miss Grace Haxton Giffen, both of the Edinburgh Medical College for Women; and third prize (£5), Miss Jessie MacLaren Macgregor, of the Edinburgh Medical School for Women.

### University of Cambridge.

Degrees.—At the congregation on June 2, the following medical degrees were conferred: M.D.: E. H. Colbeck, B.A., Caius; G. H. R. Holden, M.A., B.C., Caius. M.B. and B.C.: A. Muir, B.A., Trinity; H. Simpson, B.A., St. John's; W. B. Mercer, B.A., Caius; F. J. Gillibrand, M.A., Queen's; W. P. Fookes, B.A., Jesus.

### ATHLETICS.

### St. George's Hospital Sports.

These sports were held on June 14th at Stamford Bridge. Mr. C. T. Dent, F.R.C.S., filled the office of referee; Mr. E. B. Turner, F.R.C.S., was starter; Messrs. H. W. Allington, F.R.C.S., and C. E. Cotes, R.R.C.S., were judges; and Mr. J. S. Harvey was hon. sec.; and Mrs. Brudenell Carter presented the prizes.

100 Yards Level Race.—Heat 1: W. M. Woodhouse, 1; W. Chesnaye, 2; H. Breton, 0; J. F. H. Iles, 0. Won easily by two yards. Time, 11 2-5 sec. Heat 2: R. C. Gayer, 1; M. Breton, 2; F. H. Walker, 0; A. L. Tatham, 0. Won after a good race by half a yard. Time, 11 1-5 sec. Heat 3: C. N. Barton, w.o. Final Heat: Woodhouse, 1; M. Breton, 2; Gayer, 3; Chesnaye, 0; Barton, 0. Won by a yard and a half; a foot separated second and third. Time, 10 4-5 sec.

880 Yards Handicap.—J. C. Gardner, scratch, 1; H. M. Cooper, 10 yards start, 2; J. F. H. Iles, 25, 0; C. Chadborn, 15, 0; R. C. Gayer, 40, 0; C. N. Barton, 45, 0; A. S. Rancome, 5, 0. Cooper took the lead two hundred and twenty yards from home, and retained it until thirty yards from the winning-post, when Gardner came with a fine spurt and won a good race by half a yard. Time, 2 min. 12 1-5 sec. Only the two named finished.

Putting the Weight.—J. C. Gardner, scratch, 31 ft. 5 in., 1; M. Breton, penalised 3 ft., 32 ft. 6½ in., 2; H. Breton, penalised 1 ft., 29 ft. 10½ in., 3.

120 Yards Hurdle Race.—A. L. Tatham, 1; J. F. H. Iles, 2; J. S. Harvey, 0. Tatham got away well, and leading from start to finish scored an easy victory by ten yards from Iles. Time, 21 1-5 sec.

Long Jump.—A. L. Tatham, 18 ft. 3 in., 1; J. F. H. Iles, 18 ft. 1½ in., 2; L. A. Grimes, 13 ft. 7 in., 3.

One Mile Handicap.—H. M. Cooper, 40 yards start, 1; J. H. Hawes, scratch, 2; J. S. Harvey, 10, 0; J. F. H. Iles, 25, 0; A. S. Ransome, 15, 0. Cooper's ability had evidently been very much under-estimated by the handicapper, as he led from start to finish, and won by fully ninety yards. Only two finished. Time, 4 min. 56 2-5 sec.

Two Miles Bicycle Handicap.—W. Smith, scratch, 1; C. Chadborn, 220 yards start, 2; F. Romer, 440, 3; J. S. Harvey, 600, 0. The scratch man and Chadborn were in front 600 yards from home, and Smith drawing away in the last lap won by 40 yards; bad third. Time, 6 min. 47 1-5 sec.

220 Yards Handicap.—Heat 1: W. P. Jones, 7 yards start, 1; R. C. Gayer, 3, 2; W. Chesnaye, 7, 0. Won very easily. Time, 24 3-5 sec. Heat 2: J. F. H. Iles, 6 yards start, 1; J. Harvey, 4, 2. Won by three yards. Time, 27 4-5 sec. Heat 3: L. A. Grimes, 12 yards start, 1; W. Woodhouse, scratch, 2. Won in a canter. Time, 25 4-5 sec. Final heat: Jones, 1; Gayer, 2; Grimes, 3; Iles, 0; Woodhouse, 0. Won by a yard and a half; a bad third. Time, 24 1-5 sec.

High Jump.—L. A. Grimes, 4 ft. 8 in., 1; J. S. Harvey, 0; W. Woodhouse, 0; A. L. Tatham, 0.

440 Yards Race.—J. C. Gardner, 1; F. H. Walker, 2; W. P. Jones, 3; C. Chadborn, 0; R. C. Gayer, 0. Won by ten yards; a bad third. Time, 57 3-5 sec.

440 Yards Handicap (Open).—E. C. Bredin, L.A.C., scratch, 1; R. Grist, L.A.C., 25 yards start, 2; F. S. Evans, L.A.C., 31, 3; J. P. Shuter, 8, 0; J. G. Turner, L.A.C., 8, 0; L. A. Williams, Middlesex Hospital, 18, 0; F. A. Waylen, L.A.C., 18, 0; H. W. Dudgeon, L.A.C., 23, 0; F. H. Bailey-King, Middlesex Hospital, 23, 0; H. E. Hakin, L.A.C., 36, 0. The scratch man ran in good form, and getting in front forty yards from the tape, won by ten yards; bad third. Time, 56 4-5 sec.

A Consolation Race, Sack Race, and Tug-of-War were also included in the programme.

